# Acupuncture methods for insomnia disorder in the elderly: protocol for a systematic review and network meta-analysis

**DOI:** 10.1186/s13643-023-02287-1

**Published:** 2023-07-14

**Authors:** Weitao Dong, Hao Zhou, Rong Wu, Ximeng He, Xingliang Chen, Hongchi Zhou, Tingting Gong, Chao Wang

**Affiliations:** 1 Acupuncture and Tuina School, Chengdu University of Traditional Chinese Medicine, Chengdu, China; 2 Sichuan Integrative Medicine Hospital, Chengdu, China; 3 Department of Rheumatology Immunohematology, Affiliated Hospital of Shaanxi University of Traditional Chinese Medicine, Xianyang, China

**Keywords:** Acupuncture therapy, Elderly, insomnia disorder, Network meta-analysis (NMA)

## Abstract

**Background:**

Insomnia disorder remains one of the most common sleep disorders in the elderly, with high prevalence and substantial consequences for patients’ general health. Despite that increasing clinical trials have indicated that acupuncture seems to be effective for insomnia disorder in the elderly, comparative efficacy and safety of different acupuncture methods for elderly individuals with insomnia disorder has been unclear. Therefore, this protocol outlined a plan to evaluate and rank the efficacy and safety of various acupuncture approaches for insomnia disorder in the elderly.

**Methods:**

A systematic search of 8 bibliographic databases will be conducted from their inception to 18 June 2023, including Cochrane Library, MEDLINE (via PubMed), Embase, Web of Science, Chinese National Knowledge Infrastructure (CNKI), Wanfang Database, VIP Database, and Chinese Biomedical Literature Database (CBM). Randomized controlled trials investigating acupuncture methods for insomnia disorder in the elderly, published in English or Chinese will be included. The primary outcome is sleep quality measured by the Pittsburgh Sleep Quality Index (PSQI). Two reviewers will independently perform study selection, data extraction and risk assessment of bias. The quality of included literatures will be appraised using Cochrane risk-of-bias tool (ROB 2.0). ADDIS (Aggregate Data Drug Information System) V.1.16.8 will be used to conduct Bayesian network meta-analysis. The quality of evidence will be evaluated using the Grading of Recommendations Assessment, Development and Evaluation System (GRADE).

**Discussion:**

In this study, the results will provide credible evidence to assess the efficacy and safety of acupuncture therapies for elderly patients with insomnia disorder, assisting patients, physicians and clinical research investigators to select the most appropriate acupuncture method.

**Systematic review registration:**

The protocol has been registered at OSF (https://osf.io/3kjpq/) with a registration number https://doi.org/10.17605/OSF.IO/3KJPQ.

**Supplementary Information:**

The online version contains supplementary material available at 10.1186/s13643-023-02287-1.

## Background

Insomnia disorder is a common disease worldwide, which is one of the most common sleep disorders among the elderly [1–3]. It has been shown that more than 50% of the elderly worldwide suffer from sleep disorders, of these, 20% to 40% suffer from insomnia disorder [4]. Women have a higher incidence of insomnia disorder than men [5, 6]. The main symptom of insomnia is difficulty in falling asleep or sleep maintenance disorder [7]. However, long-term insomnia disorder will not only increase the risk of depression and death in the elderly, but also is an independent risk factor for cardiovascular disease and diabetes [8–12]. which seriously affects the quality of life of patients and causes greater psychological and economic burden to patients and their families, and insomnia disorder has become a major public health problem [13–15].

Treatment of insomnia disorder can be divided into pharmacological and non-pharmacological interventions [16]. Benzodiazepine receptor agonists as pharmacological interventions have the most evidence for treating insomnia, with serious side effects such as increased risks of falls and hip fractures, over-sedation, and confusion among the elderly [17–21]. Therefore, several authors propose that non-pharmacological interventions should be considered as first-line treatment options for insomnia disorder in the elderly [22]. Among non-pharmacological interventions, cognitive behavioral therapy (CBT) has been recognized as a first-line treatment for insomnia disorder [23]. However, high costs and lack of professionals limit its availability [24]. Consequently, for the elderly, seeking for an effective, simple, safe treatment of insomnia disorder appears to have great significance.

Acupuncture, as an important part of complementary and alternative therapies, has numerous advantages, such as wide indication, reliable curative effect, convenient operation, economic safety, and has been widely accepted and applied worldwide [25]. Acupoints on the meridians are stimulated by using disposable sterile acupuncture needles [26]. A growing number of randomized controlled trials (RCT) have indicated that acupuncture could improve sleep quality, prolong sleep time, reduce the severity of insomnia disorder among the elderly [27–29]. Clinically, multiple acupuncture methods are available for insomnia disorder in the elderly, such as manual acupuncture, electroacupuncture, auricular acupuncture, and auricular acupressure. However, due to the diversity of acupuncture approach, determining the most effective acupuncture method is intractable, which makes clinicians confused about how to select the optimal acupuncture method for elderly individuals with insomnia disorder.

Up to now, among appraising multiple competing interventions, network meta-analysis is considered as precisely effective approach, which can provide the rankings of distinctive acupuncture methods [30]. Therefore, the objective of this systematic review and network meta-analysis is to generate a clinically useful evidence-based hierarchy for efficacy and safety of various acupuncture approaches for insomnia disorder in the elderly.

## Methods

The protocol was performed in compliance with the Preferred Reporting Items for Systematic Reviews and Meta-Analyses Protocols (PRISMA-P) statement and the Checklist of Items to Include When Reporting a Systematic Review Involving a Network Meta-analysis [31, 32] (Additional file 1). Meanwhile, it has been registered with OSF with registration number https://doi.org/10.17605/OSF.IO/3KJPQ.

### Criteria for including studies in this review

#### Types of studies

RCTs reported in English or Chinese without any regional restrictions will be included. In case of a randomized crossover trial, we will include only the first-phase results. Animal experimental studies, quasi-RCTs, case report, expert experience, conference article and duplicated publications will be excluded.

#### Types of patients

Elderly patients (≥ 60 years) who have been diagnosed with insomnia disorder, according to the Diagnostic and Statistical Manual of Mental Disorders (DSM) [33], the International Classification of Sleep Disorders (ICSD) [34], the Chinese Classification of Mental Disorders (CCMD) [35] or other accepted diagnostic standards will be included, regardless of gender, race, economic status, ethnicity or the severity of insomnia disorder. Patients with drug allergies or other serious medical conditions such as cancer, liver disease, or kidney disease should be excluded from the original trials.

#### Types of interventions

Acupuncture will be defined in this review as acupoint-based therapy, irrespective of needling techniques and stimulation method, including manual acupuncture, electro-acupuncture, auricular acupuncture, auricular acupressure, acupressure, acupoint application, moxibustion, catgut embedding, transcutaneous electrical acupoint stimulation, acupoint injection, and others. Interventions without stimulating the acupoint will be ruled out.

#### Types of control groups

Treatments in the control groups will include sham acupuncture, placebo, pharmacotherapy, no treatment, waiting list, lifestyle modifications, or usual care. Studies that compared the distinctive types of acupoint-based therapy will be included.

#### Types of outcome measures

Studies that report one or more of the below-mentioned outcomes will be included. Otherwise, the trial will be excluded.

#### Primary outcomes

The review will mainly aim to investigate various acupuncture methods in the improvement of the sleep quality. Therefore, the change of the sleep quality will be measured by the Pittsburgh Sleep Quality Index (PSQI) [36].

#### Secondary outcomes

The secondary outcomes will include: (I) sleep onset latency (in min) and sleep efficiency (%). Sleep efficiency is defined as the total sleep time, divided by the total recording time; (II) Insomnia Severity Index [37]; (III) Quality of Life Scale (SF-36) [38]; and (IV) adverse events measured by the Treatment Emergent Symptom Scale (TESS) [39] or the incidence of adverse events.

### Search methods for identification of studies

#### Electronics searches

The following eight electronic databases including Cochrane Library, MEDLINE (via PubMed), Embase, Web of Science, Chinese National Knowledge Infrastructure (CNKI), Wanfang Database, VIP Database, and Chinese Biomedical Literature Database (CBM) will be searched from their inception to 18 June 2023. Gray literature should also be searched. All randomized controlled trials reported in English or Chinese will be included. In addition, qualified research conference abstracts, reference lists of manuscripts and trial registry database (WHO International Clinical Trials Registry Platform and Clinical Trials. gov) will be retrieved to identify additional studies. The search strategy consists of Medical Subject Headings (MeSH) and free-text terms, such as insomnia OR insomnia disorder AND aged OR elderly AND acupuncture OR acupuncture therapy OR manual acupuncture OR electroacupuncture AND randomized. The proposed detailed search strategy for PubMed is presented in Table 1. Detailed information on the full search strategies for all databases is available in the online Supplemental appendix 1.

**Table 1:** search strategy in PubMed database

No	Search items
#1	Acupuncture. Mesh
#2	Acupuncture therapy. ti. ab
#3	Acupuncture points. ti. ab
#4	Manual acupuncture. ti. ab
#5	Electroacupuncture. ti. ab
#6	Auricular acupuncture. ti. ab
#7	Auricular acupressure. ab
#8	Scalp acupuncture. ti. ab
#9	Body acupuncture. ti. ab
#10	1 or 2–9
#11	Randomized controlled trial. Mesh
#12	Controlled clinical trial. ti. ab
#13	Randomized. ti. ab
#14	Randomly. ti. ab
#15	Trial. ti. ab
#16	11 or 12–15
#17	Aged. Mesh
#18	Elderly. ti. ab
#19	Geriatric. ti. ab
#20	Old aged. ti. ab
#21	Aging. ti. ab
#22	17 or 18–21
#23	Insomnia. Mesh
#24	Sleeplessness. ti. ab
#25	Insomnia Disorder. ti. ab
#26	Insomnias. ti. ab
#27	Sleep Initiation and Maintenance Disorders. ti. ab
#28	Disorders of Initiating and Maintaining Sleep. ti. ab
#29	Sleep Initiation Dysfunction. ti. ab
#30	Dysfunction, Sleep Initiation. ti. ab
#31	Dysfunctions, Sleep Initiation. ti.ab
#32	Sleep Initiation Dysfunctions. ti.ab
#33	23 or 24–32
#33	#10 and #16 and #22 and #33

#### Selection of studies

**Fig. 1:**
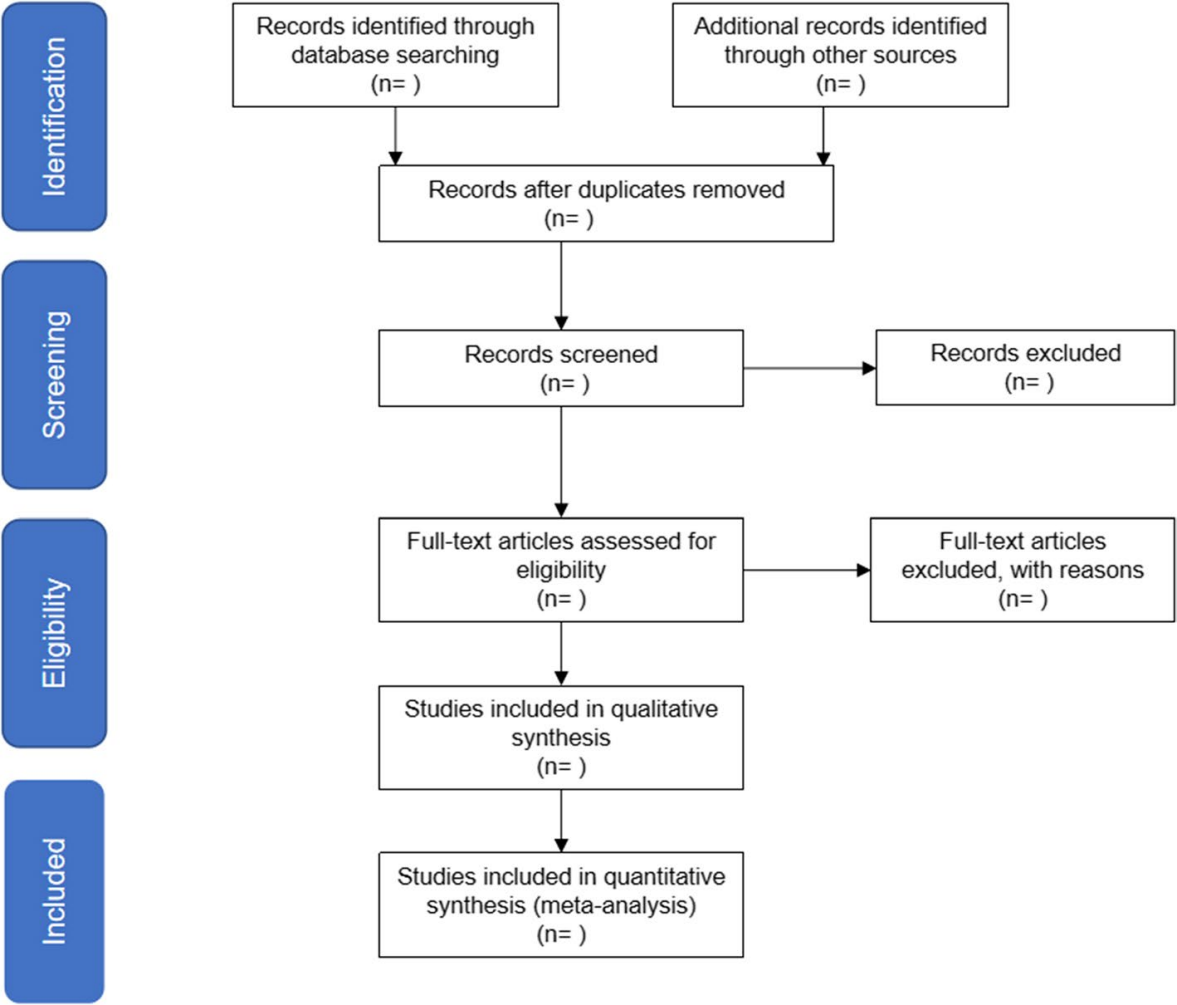
The PRISMA flow diagram of the study selection process

According to the exclusion and inclusion criteria, studies imported into Endnote X9 software after exclusion of duplicated articles will be independently screened by two authors (WD and HZ). Full texts of the relevant study will be reviewed to confirm the final inclusion of studies. For unclear study, the author will be contacted for details to determine whether this literature would be included. Any disagreement on articles will be resolved through discussion or rechecked by a third reviewer (TG) until consensus will be reached. The outline of the study selection procedure will be presented in a Preferred Reporting Items for Systematic Reviews and Meta-Analyses flow chart Fig. 1.

#### Data extraction and management

Based on a self-designed data acquisition form, 2 reviewers (RW and XH) will independently extract the following information from eligible studies: (I) general information (country, publication year, first author, study type, number of centers, sample size and study design); (II) patient information (age, gender, ethnicity, diagnosis, insomnia intensity before treatment); (III) intervention and comparator (types of acupuncture, selection of acupuncture points and treatment frequency /session/duration, follow-up period,); (IV) outcomes (data and time points for each measurement, types of outcomes); (V) adverse events; for missing data or clarification for unclear information, an effort will be made to contact the corresponding author to obtain detailed information. In case of ambiguity and divergence, it will be resolved through discussion or the involvement of the third reviewer (CW). Finally, the data will be transferred to RevMan software.

#### Quality assessment

The quality of the included studies will be evaluated using the Cochrane Risk of Bias 2 tool (RoB2) [40] by two independent reviewers (XC and HZ) according to the below-mentioned five domains:bias arising from the randomization process; bias due to deviations from intended interventions; bias due to missing outcome data; bias in measurement of the outcome and bias in selection of the reported result. The risk of bias assessed by each outcome will be judged (low/high/some concerns) and the overall risk of bias of the assessment results will be predicated. Any discrepancy will be arbitrated by a third reviewer (TG). If a RCT was judged to have “some concerns” about the risk of bias in three or more domains, it will be excluded from the systematic review due to the high risk of bias.

The Grading of Recommendations Assessment, Development, and Evaluation (GRADE) system [41] will be applied to evaluate the strength of the evidence by 2 independent reviewers (WD and HZ). Evidence quality will be judged as “high-quality”, “moderate quality”,“low quality”, or “very low quality” in terms of the risk of bias, inconsistency, indirectness, imprecision and publication bias. If any dispute occurs, the third reviewer (CW) will act as a referee.

#### Assessment of similarity and consistency

In order to acquire a credible and valid result, the assessment of similarity and consistency will be carried out. Due to the difficulty in determining similarity through statistical analysis, the assessment will be performed based on clinical and methodological characteristics, including study designs (risk of bias), patient characteristics (age and severity of insomnia), and interventions (duration/types/sessions/intensity of treatment and needling techniques). Both a local and global approach will be applied to assess the assumption of consistency. The local consistency of direct and indirect evidence will be evaluated using the node-splitting approach and we will calculate the *P* value [42]. If the node shows a *P* value greater than 0.05, there is no significant statistical difference; conversely, the comparison of direct and indirect evidence will be considered as inconsistency [43]. In the meantime, inconsistency throughout the entire network will be assessed applying the design-by-treatment interaction model and chi-squared test [44].

### Statistical analysis

#### Pairwise meta-analysis

The premise of pairwise meta-analysis is law of similarity, which means only when the included studies meet a certain degree of similarity, can the meta-analysis be carried out. We stipulate that no less than 3 studies of same interventions and outcome indicators can make sense in pairwise meta-analysis. The software RevMan version 5.4 (Review Manager, The Cochrane Collaboration, 2020) will be applied to conduct Pairwise meta-analysis to compare treatments with direct evidence. The effect sizes will be calculated employing the change from baseline in sleep quality. Standardized mean differences (SMD) for continuous outcomes or ORs for dichotomous outcomes, both with a 95% CI, will be calculated as effect measures. The Cochran’s *Q* χ^2^ test and *I*^2^ statistic will be measured for heterogeneity as a measure to reflect the underlying differences between the RCTs that directly compare the same pair of interventions. A *p* value of up to 0.10, and *I*^2^ value of above 50% will indicate high heterogeneity. Sensitivity analysis of pairwise meta-analyses will be performed to validate the robustness of the results by omitting studies with unacceptable sources of heterogeneity.

#### Network meta-analysis

The network meta-analysis will be conducted using Aggregate Data Drug Information System (ADDIS) V.1.16.8 software (Drugis, Groningen, NL) [43, 45]. A random effects model will be applied to perform a Bayesian network meta-analysis to integrate the direct and indirect evidence with the Markov Chain Monte Carlo method. Four chains will be applied as parameters for simulation. There are 50,000 simulation iterations in each chain, and the researcher will discard the first 20,000 simulations to remove the effect of the initial value. Visual inspection of the trace plots will be used to appraise model convergence and taking the Gelman-Rubin statistic into consideration. In the meantime, the network diagram will be generated using STATA V.15.0 software (Stata Corp., College Station, TX, USA) and compare each outcome. In light of each specific outcome, the effects of different acupuncture approaches will be sequenced to display the most effective surface under the cumulative ranking curve and mean ranks with their 95% CI.

#### Assessment of heterogeneity

The Q test and I^2^ statistics will be applied to calculate statistical heterogeneity among studies. In the light of the Cochrane Handbook [46], The *I*^2^ value is rated as the following four levels: little or no heterogeneity (0–40%), moderate heterogeneity (30–60%), substantial heterogeneity (50–90%) and considerable heterogeneity (75–100%). To explore potential sources of heterogeneity from the clinical and methodological perspective, subgroup or sensitivity analysis will be performed.

#### Meta-regression, subgroup analysis, and sensitivity analysis

IF feasible, meta-regression and subgroup analysis will also be performed, to detect the possible sources of inconsistency and heterogeneity. Meta-regression will be conducted with the following covariates: (I) initial severity of the disease; (II) sample size; (III) average age; and (IV) the duration, frequency, and course of acupuncture methods. Subgroup analyses will be employed based on the different comparators and various forms of acupuncture methods. Multiple sensitivity analyses for the analyzed outcomes will be performed to assess the findings’ robustness and examine if any particular study accounted for a large proportion of heterogeneity. The analyses will be based on different statistical models and qualities of methodology. The meta-analysis will be repeated, and more inferior quality studies will be excluded.

### Assessment of publication bias

A comparison-adjusted funnel plot will be applied to inspect reported bias and small-scale effects visually. If more than 10 trials are available, the funnel plot will be employed to assess the reported bias [47]. If the funnel plot is found to be asymmetrical, Egger’s regression test will be used to analyze the causes of asymmetry. All eligible trials, regardless of the quality of their methodologies, will be included.

### Ethics and informed consents

No ethics approval is required since this systematic review and network meta-analysis do not collect confidential personal data and do not perform interventions in treating patients. Besides, the findings will be disseminated through a peer-reviewed journal.

## Discussion

It is reported that various acupuncture methods have been proved to be effective for insomnia disorder in the elderly. However, due to high variability in selecting these acupuncture approaches for clinicians, the Bayesian network meta-analysis will be applied to compare not only the efficacy of available acupuncture therapies, but also rank the probability of acupuncture methods regarding each domain of insomnia disorder in the elderly. To the best of our knowledge, the protocol will be the first systemic review (SR) and NMA to examine acupuncture methods for elderly individuals with insomnia disorder. We sincerely hope that the findings could assist patients, physicians, and clinical research investigators to making more informed treatment decisions. Admittedly, it is worth noting that there may be some limitations existing in this review. On the one hand, only Chinese and English studies were retrieved because of language barrier, which may result in bias. On the other hand, our proposed methodology will only place emphasis on acupuncture approaches, whereas less attention will be paid to the analysis of specific details regarding acupuncture application or acupuncture points selection. The results will be disseminated through peer-reviewed journals or conference reports. The protocol will be updated quickly when supplements are required.

## Supplementary Information

**Additional file 1.** PRISMA-P 2015 Checklist.

**Additional file 2:** **Supplemental Appendix 1**.

## Data Availability

Not applicable.

## Abbreviations

PRISMA-P: Preferred Reporting Items for Systematic Reviews and Meta-Analysis Protocols
RCT: Randomized controlled trial
MD: Mean difference
CI: Confidence interval
SMD: Standardized mean difference
RR: Relative risk
OR: Odds ratio
