# Supplementary material for: Acupuncture methods for insomnia disorder in the elderly: protocol for a systematic review and network meta-analysis
Source: Syst Rev. 2023 Jul 14;12:124. doi: 10.1186/s13643-023-02287-1 (PMC10347792; doi:10.1186/s13643-023-02287-1)
Supplement: Supplementary file 2 — Additional file 2: Supplemental Appendix 1. [file 13643_2023_2287_MOESM2_ESM.doc]

**1.** Search strategy in PubMed database

| No. Search items |
| --- |
| #1 Acupuncture. Mesh.  #2 Acupuncture therapy. ti. ab.  #3 Acupuncture points. ti. ab.  #4 Manual acupuncture. ti. ab.  #5 Electroacupuncture. ti. ab.  #6 Auricular acupuncture. ti. ab.  #7 Auricular acupressure. ab.  #8 Scalp acupuncture. ti. ab.  #9 Body acupuncture. ti. ab.  #10 1 or 2-9  #11 Randomized controlled trial. Mesh.  #12 Controlled clinical trial. ti. ab.  #13 Randomized. ti. ab.  #14 Randomly. ti. ab.  #15 Trial. ti. ab.  #16 11 or 12-15  #17 Aged. Mesh.  #18 Elderly. ti. ab.  #19 Geriatric. ti. ab.  #20 Old aged. ti. ab.  #21 Aging. ti. ab.  #22 17 or 18-21  #23 Insomnia. Mesh.  #24 Sleeplessness. ti. ab.  #25 Insomnia Disorder. ti. ab.  #26 Insomnias. ti. ab.  #27 Sleep Initiation and Maintenance Disorders. ti. ab.  #28 Disorders of Initiating and Maintaining Sleep. ti. ab.  #29 Sleep Initiation Dysfunction. ti. ab.  #30 Dysfunction, Sleep Initiation. ti. ab.  #31 Dysfunctions, Sleep Initiation. ti.ab.  #32 Sleep Initiation Dysfunctions. ti.ab.  #33 23 or 24-32  #33 #10 and #16 and #22 and #33 |

**2.** Search strategy in Embase

| No. Search items |
| --- |
| #1 'Acupuncture'/exp  #2 'Acupuncture therapy':ti,ab,kw OR 'Acupuncture points':ti,ab,kw OR 'Manual acupuncture':ti,ab,kw OR 'Electroacupuncture':ti,ab,kw OR  'Auricular acupuncture':ti,ab,kw OR 'Auricular acupressure':ti,ab,kw OR 'Scalp acupuncture':ti,ab,kw OR 'Body acupuncture':ti,ab,kw  #3 #1 OR #2  #4 'Randomized controlled trial'/exp  #5 'Controlled clinical trial':ti,ab,kw OR 'Randomized':ti,ab,kw OR 'Randomly':ti,ab,kw  #6 #4 OR #5  #7 'Aged'/exp  #8 'Elderly':ti,ab,kw OR 'Geriatric':ti,ab,kw OR 'Old aged':ti,ab,kw OR 'Aging':ti,ab,kw  #9 #7 OR #8  #10 'Insomnia'/exp  #11 'Sleeplessness':ti,ab,kw OR 'Insomnia Disorder':ti,ab,kw OR 'Insomnias':ti,ab,kw OR 'Sleep Initiation and Maintenance Disorders':ti,ab,kw OR 'Disorders of Initiating and Maintaining Sleep':ti,ab,kw OR 'Sleep Initiation Dysfunction':ti,ab,kw OR 'Dysfunction, Sleep Initiation':ti,ab,kw OR 'Dysfunctions, Sleep Initiation':ti,ab,kw OR 'Sleep Initiation Dysfunctions':ti,ab,kw  #12 #9 OR #10  #13 #3 AND #6 AND #9 AND #12 |

**3.** Search strategy in Web of Science

| No. Search items |
| --- |
| #1 Topic:(“Acupuncture” OR “Acupuncture therapy” OR “Acupuncturepoints” OR “Manual acupuncture” OR “Electroacupuncture” OR “Auricular acupuncture” OR “Auricular acupressure” OR “Scalp acupuncture” OR “Body acupuncture”  #2 “Randomized controlled trial” OR “Controlled clinical trial” OR “Randomized” OR “Randomly”  #3 “Aged” OR “Elderly” OR “Geriatric” OR “Old aged” OR “Aging”  #4 “Insomnia” OR “Insomnia Disorder” OR “Sleeplessness” OR “Insomnias” OR “Sleep Initiation and Maintenance Disorders” OR “Disorders of Initiating and Maintaining Sleep” OR “Sleep Initiation Dysfunction” OR “Dysfunction, Sleep Initiation” OR “Dysfunctions, Sleep Initiation” OR “Sleep Initiation Dysfunctions”  #5 #1 AND #2 AND #3 AND #4 |

**4.** Search strategy in Cochrane Library

| No. Search items |
| --- |
| #1 MeSH descriptor: [Acupuncture] explode all trees  #2 (Acupuncture therapy):ti,ab,kw OR (Acupuncture points):ti,ab,kw OR (Manual acupuncture):ti,ab,kw OR (Electroacupuncture):ti,ab,kw OR (Auricular acupuncture):ti,ab,kw OR (Auricular acupressure):ti,ab,kw OR (Scalp acupuncture):ti,ab,kw OR (Body acupuncture):ti,ab,kw  #3 #1 OR # 2  #4 MeSH descriptor: [Randomized controlled trial] explode all trees  #5 (Controlled clinical trial):ti,ab,kw OR (Randomized):ti,ab,kw OR (Randomly):ti,ab,kw  #6 #4 OR # 5  #7 MeSH descriptor: [Aged] explode all trees  #8 (Elderly):ti,ab,kw OR (Geriatric):ti,ab,kw OR (Old aged):ti,ab,kw OR (Aging):ti,ab,kw  #9 #7 OR # 8  #10 MeSH descriptor: [Insomnia] explode all trees  #11 (Insomnia Disorder):ti,ab,kw OR (Sleeplessness):ti,ab,kw OR (Insomnias):ti,ab,kw OR (Sleep Initiation and Maintenance Disorders):ti,ab,kw OR (Disorders of Initiating and Maintaining Sleep):ti,ab,kw OR (Sleep Initiation Dysfunction):ti,ab,kw OR (Dysfunction, Sleep Initiation):ti,ab,kw OR (Dysfunctions, Sleep Initiation):ti,ab,kw OR (Sleep Initiation Dysfunctions):ti,ab,kw  #12 #10 OR # 11  #13 #3 AND # 6 AND #9 AND #12 |

**5.** Search strategy in China National Knowledge Infrastructure Database

| No. Search items |
| --- |
| #1 SU=(针刺'+'电针'+'耳针'+'耳穴按压'+'头针'+'手针'+'体针')  #2 SU='老年失眠障碍'  #3 #1 AND # 2 |

**6.** Search strategy in Wan Fang Data

| No. Search items |
| --- |
| #1 主题=(针刺or电针or耳针or耳穴按压or头针or手针or 体针)  #2 主题=(老年失眠障碍)  #3 #1 AND # 2 |

**7.** Search strategy in VIP Chinese Science and Technology Periodical Database

| No. Search items |
| --- |
| #1 M=(针刺or电针or耳针or耳穴按压or头针or手针or 体针)  #2 M=(老年失眠障碍)  #3 #1 AND # 2 |

**8.** Search strategy in Chinese Biomedical Literature Database

| No. Search items |
| --- |
| #1 常用字段=(针刺or电针or耳针or耳穴按压or头针or手针or 体针)  #2 常用字段=(老年失眠障碍)  #3 #1 AND # 2 |

1. **ClinicalTrials.gov search strategy**

Available, Completed Studies | Studies With Results | Interventional Studies | Insomnia | Studies with elderly Participants

1. **the WHO International Clinical Trials Registry Platform search strategy**

Insomnia in the title AND acupuncture therapy in the Condition AND with results only.
